# Extracorporeal CO_2_ removal integrated with continuous renal replacement therapy in patients with acute respiratory distress syndrome and acute kidney injury: a systematic review and meta-analysis

**DOI:** 10.1016/j.aicoj.2026.100114

**Published:** 2026-07-15

**Authors:** Yuri de Albuquerque Pessoa dos Santos, Paulo Ricardo Gessolo Lins, Bruno Martins Tomazini, Eduardo Lyra de Queiroz, Laerte Pastore Júnior, Fernando José da Silva Ramos, Maristela Carvalho de Costa, Eduardo Leite Vieira Costa, Marcelo Brito Passos Amato, Alain Combes

**Affiliations:** a Intensive Care Unit, Hospital Sírio-Libanês, Rua Dona Adma Jafet, 91, Bela Vista, São Paulo, SP, 01308-050, Brazil; b Federal University of São Paulo (UNIFESP), Rua Napoleão de Barros, 715, Vila Clementino, São Paulo, SP, 04024-002, Brazil; c Research Institute, Hospital do Coração, Rua Desembargador Eliseu Guilherme, 147, Paraíso, São Paulo, SP, 04004-030, Brazil; d Nephrology Service, Heart Institute (InCor), University of São Paulo, Av. Dr. Enéas de Carvalho Aguiar, 44, São Paulo, SP, 05403-000, Brazil; e Respiratory Intensive Care Unit, School of Medicine, University of São Paulo, Av. Dr. Arnaldo, 455, Cerqueira César, São Paulo, SP, 01246-903, Brazil; f Sorbonne Université, INSERM, UMRS_1166-ICAN, Institute of Cardiometabolism and Nutrition, Assistance Publique–Hôpitaux de Paris, Sorbonne Université Hôpital Pitié–Salpêtrière, 47, Boulevard de l’Hôpital, Paris F-75013, France

**Keywords:** Acute respiratory distress syndrome, Acute kidney injury, Continuous renal replacement therapy, Hypercapnia, Positive-pressure respiration

## Abstract

**Introduction:**

Patients with acute respiratory distress syndrome (ARDS) and acute kidney injury (AKI) requiring continuous renal replacement therapy (CRRT) represent a complex clinical scenario. Permissive hypercapnia during lung-protective ventilation may cause severe acidemia and limit further reductions in ventilatory intensity. Extracorporeal carbon dioxide removal integrated into the CRRT circuit may enhance carbon dioxide clearance while providing renal support, but its physiological effects and safety in this population remain uncertain.

**Methods:**

We conducted a systematic review and meta-analysis of adult studies evaluating ECCO_2_R integrated with continuous RRT in patients with ARDS and AKI. Literature searches were performed in PubMed, Embase (Ovid), and Cochrane CENTRAL from inception to May 15, 2026, without language or date restrictions, and were supplemented by reference screening, forward citation tracking, and trial registry searches. The primary outcomes were changes in PaCO_2_ and arterial pH after treatment initiation. Secondary outcomes included changes in ventilatory parameters, clinical outcomes, and safety events. Pre-post changes were pooled using random-effects models with restricted maximum likelihood estimation.

**Results:**

Seven observational studies comprising 105 patients were included. ECCO_2_R integrated with RRT was associated with sustained reductions in PaCO_2_ at 2 h (mean difference [MD] −8.22 mmHg; 95% CI −12.00 to −4.44), 6 h (MD −9.02 mmHg; 95% CI −15.69 to −2.34), and 24 h (MD −9.10 mmHg; 95% CI −13.59 to −4.61). These changes were accompanied by increases in arterial pH at 2 h (MD + 0.05; 95% CI 0.02 to 0.08) and 24 h (MD + 0.06; 95% CI 0.02 to 0.11). Ventilatory variables also improved, with reductions in tidal volume normalized to predicted body weight (MD −1.14 mL/kg PBW; 95% CI −1.74 to −0.54), driving pressure (MD −3.61 cmH_2_O; 95% CI −4.87 to −2.35), and mechanical power (MD −6.82 J/min; 95% CI −9.12 to −4.51). Safety and patient-centered outcomes were limited and heterogeneously reported.

**Conclusion:**

ECCO_2_R integrated with CRRT was associated with early PaCO_2_ reduction, pH improvement, and lower ventilatory intensity in patients with ARDS and AKI. These findings support the physiological feasibility of this combined approach, although prospective comparative studies are needed to define its clinical role, safety, and impact on patient-centered outcomes.

**Trial registration:**

PROSPERO CRD420251234033.

## Highlights

• Seven studies assessed ECCO_2_R-CRRT in ARDS with concomitant AKI.
• ECCO_2_R-CRRT reduced PaCO_2_ and improved arterial pH within 24 h.
• Driving pressure, tidal volume, and mechanical power decreased after support
• Circuit clotting predominated, whereas bleeding events were rare
• Findings support the physiological feasibility of combined extracorporeal support

## Graphical abstract

**Figure:**
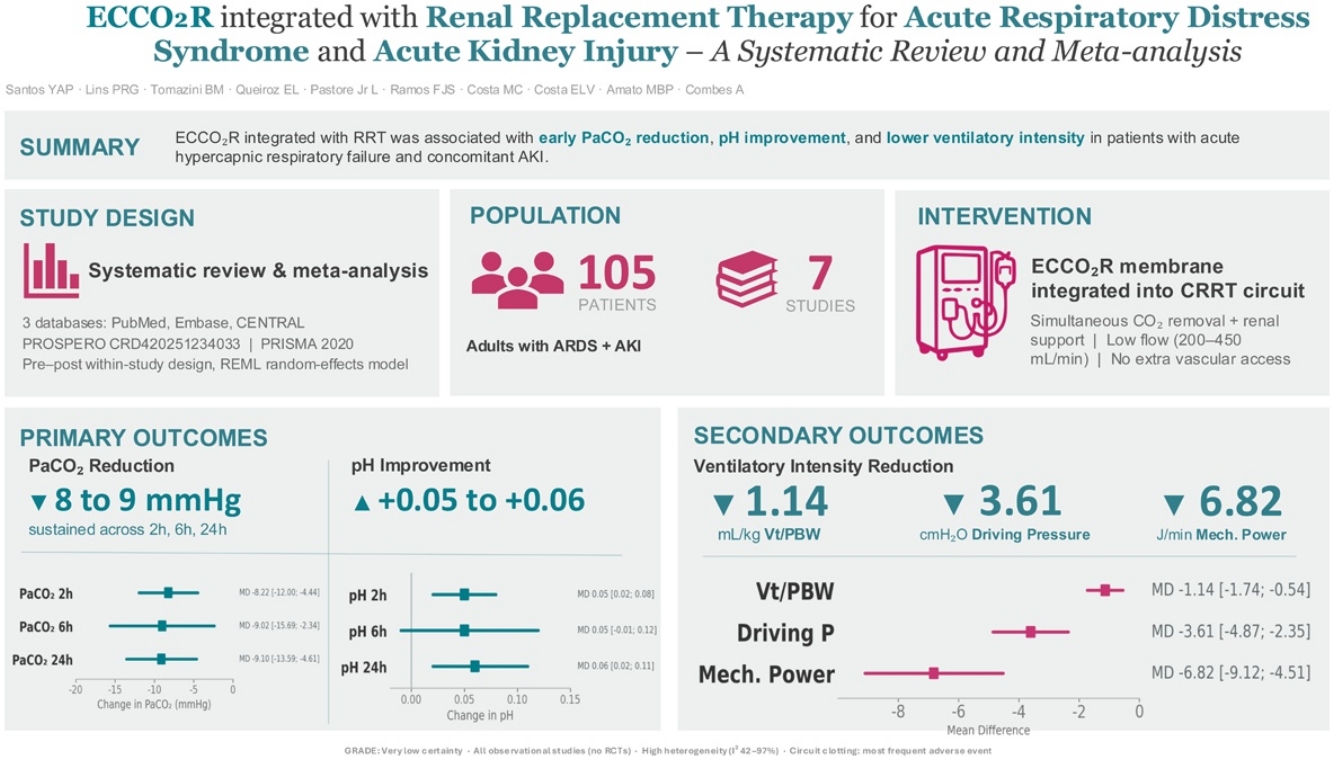

## Introduction

Acute respiratory distress syndrome (ARDS) is a major cause of acute hypoxemic respiratory failure in critically ill adults and is frequently complicated by acute kidney injury (AKI). Lung-protective ventilation and permissive hypercapnia are established strategies to mitigate ventilator-induced lung injury (VILI), while contemporary management increasingly emphasizes reductions in tidal volume (Vt), driving pressure (ΔP), and mechanical power (MP) to limit ventilatory intensity and mechanical energy transfer to the injured lung [[1], [2], [3], [4], [5]]. However, further reductions in ventilatory support may be constrained by respiratory acidosis, particularly when renal dysfunction coexists [6,7]. In patients with AKI, impaired renal acid–base regulation limits metabolic compensation for hypercapnia, favoring the development of mixed acidosis. This interaction between lung injury, hypercapnia, and kidney dysfunction may increase physiological instability and has been associated with prolonged mechanical ventilation and worse outcomes [8,9].

The quest for "protective ventilation" has led to the development of extracorporeal carbon dioxide removal (ECCO_2_R). Initial studies provided some optimism; the Xtravent trial suggested a reduction in ventilator-free days in patients with PaO_2_/FiO_2_ <150 mmHg, while the SUPERNOVA study demonstrated the feasibility of ECCO_2_R in achieving significant reductions in ventilator intensity [10,11]. Nevertheless, the recent REST trial, the largest randomized controlled trial to date using standalone high-flow ECCO_2_R, was terminated early for futility. It failed to reduce 90-day mortality and was associated with a higher incidence of adverse events, particularly bleeding, while also failing to effectively lower PaCO_2_ or significantly impact mechanical power in the intervention group [[12], [13], [14], [15]].

To overcome the complications and technical demands of standalone high-flow systems, the integration of ECCO_2_R into renal replacement therapy (RRT) circuits has emerged as a promising hybrid approach. By leveraging standard dual-lumen hemodialysis catheters and dialysis-compatible blood flows, this low-flow approach circumvents the requirement for large-bore cannulation, potentially enhancing the safety profile and simplifying bedside implementation for patients already requiring renal support [[16], [17], [18], [19]]. However, despite its theoretical advantages and growing clinical adoption, the available evidence remains fragmented across small physiological studies and pilot experiences. Significant heterogeneity exists regarding device configurations, and the true magnitude of its physiological effect on gas exchange and its ability to facilitate ultra-protective ventilation remains unclear.

Therefore, we performed a systematic review and meta-analysis to evaluate ECCO_2_R integrated into RRT in adults with ARDS and concomitant AKI. Our primary aim was to quantify its effects on PaCO_2_ and arterial pH. Secondary aims were to assess its ability to facilitate more protective ventilation through reductions in tidal volume, driving pressure, and mechanical power, and to summarize the reported safety and technical feasibility of this hybrid approach.

## Methods

This systematic review and meta-analysis was prospectively registered in the International Prospective Register of Systematic Reviews (PROSPERO; registration number (CRD420251234033) and conducted in accordance with the *Cochrane Handbook for Systematic Reviews of Interventions* [20] and the *Preferred Reporting Items for Systematic Reviews and Meta-Analyses (PRISMA) 2020* guidelines [21].

## Eligibility criteria

We included studies enrolling adults (≥ 18 years) with acute respiratory distress syndrome and concomitant acute kidney injury requiring continuous renal replacement therapy (CVVH, CVVHD, or CVVHDF), in whom a membrane lung for ECCO_2_R was integrated into the RRT circuit. We excluded pediatric studies, case reports or case series with fewer than five patients, conference abstracts without extractable data, animal or benchtop studies, studies evaluating standalone ECCO_2_R, and studies in which ECMO was the primary extracorporeal support.

## Search strategy

A comprehensive literature search was performed in PubMed, Embase (Ovid), and the Cochrane Central Register of Controlled Trials (CENTRAL) from inception to May 15, 2026, without language or date restrictions. The search strategy combined controlled vocabulary (e.g., MeSH and Emtree terms) and free-text terms related to extracorporeal carbon dioxide removal, renal replacement therapy, acute hypercapnic respiratory failure, and acute kidney injury. In addition to database searching, we screened the reference lists of included studies and relevant reviews, performed forward citation tracking, and searched trial registries for ongoing or unpublished studies. The full search strategy is provided in Supplementary Table S1.

## Study selection

**Fig. 1:**
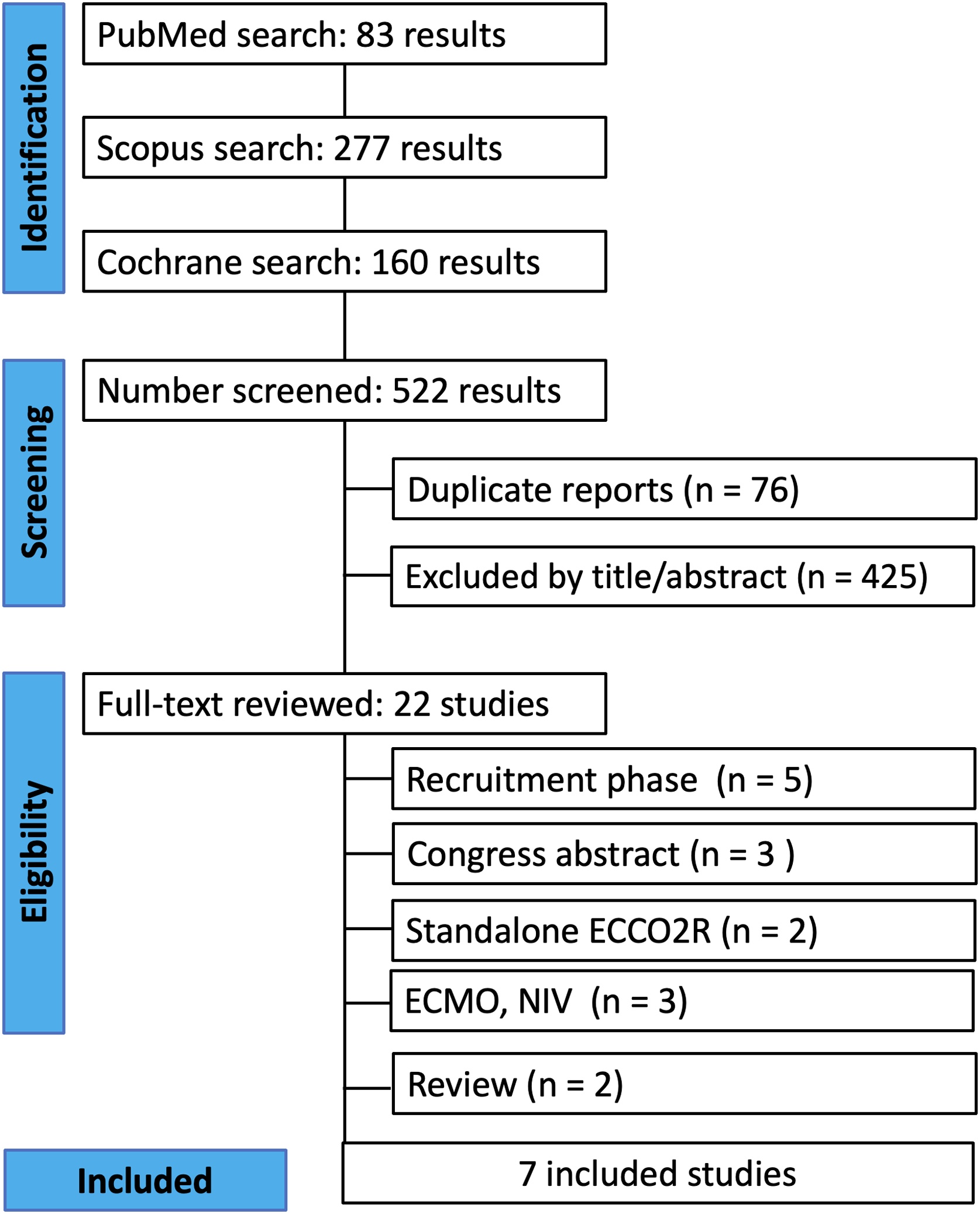
Prisma flow diagram of study screening and selection.

Two reviewers independently screened titles and abstracts and subsequently assessed full-text articles for eligibility according to predefined inclusion and exclusion criteria. Disagreements were adjudicated by a third reviewer. The study selection process is illustrated in the PRISMA 2020 flow diagram (Fig. 1).

## Data extraction

Two reviewers independently extracted data using a standardized data collection form. Extracted variables included study design, year, setting, sample size, patient characteristics, extracorporeal support configuration, gas exchange variables, ventilatory parameters, and reported clinical outcomes. When required, corresponding authors were contacted to clarify missing or incomplete data. Any disagreements were resolved by consensus.

## Risk of bias assessment

Risk of bias was assessed according to study design. Because most included studies were uncontrolled observational before-after studies, their methodological quality was evaluated using the NIH Quality Assessment Tool for Before-After (Pre-Post) Studies With No Control Group [22,23]. The only comparative non-randomized study was assessed using ROBINS-I. Two reviewers independently performed the assessments, and disagreements were resolved by a third reviewer. The results are summarized in Supplementary Table S2.

## Certainty of evidence

To evaluate the overall certainty of the evidence for each outcome, we applied the GRADE (Grading of Recommendations Assessment, Development and Evaluation) framework. Factors considered included study limitations (risk of bias), inconsistency, indirectness, imprecision, and publication bias. Each outcome was rated as having high, moderate, low, or very low certainty. GRADE assessments were based on the consensus of two authors and summarized in Supplementary Table S3.

## Outcomes

The primary outcomes of this meta-analysis were the changes in arterial partial pressure of carbon dioxide (PaCO_2_) and arterial pH after initiation of ECCO_2_R integrated with RRT, as these variables most directly reflect the early physiological effects of extracorporeal carbon dioxide clearance and correction of hypercapnic acidemia. Prespecified analyses were performed at 2, 6, and 24 h after treatment initiation, according to data availability.

Secondary outcomes comprised changes in ventilatory variables associated with ventilatory intensity and lung-protective ventilation, specifically driving pressure (ΔP), tidal volume normalized to predicted body weight (Vt/PBW), and mechanical power (MP). MP was calculated only for studies and time points with sufficient ventilatory data. For these analyses, tidal volume was converted to liters and airway pressures were expressed in cmH_2_O, so that MP was reported in J/min. When peak airway pressure (Ppeak) was available, the resistive component was incorporated into the calculation. When this variable was not reported, MP was estimated using the simplified equation based on the elastic-static and elastic-dynamic components of the respiratory system: MP = 0.098 × Vt × RR × (Pplat − 0.5 × ΔP) [4,24].

Safety and other clinical outcomes, including bleeding events, thrombocytopenia, hemolysis, circuit clotting, transfusion requirements, cannulation- or device-related complications, mortality, and ventilator-free days, were extracted when reported and summarized descriptively because of heterogeneity in outcome definitions, reporting, and patient populations across studies.

## Data synthesis and statistical analysis

Characteristics of the included studies were summarized descriptively. Continuous variables were reported as mean ± standard deviation (SD) or median [interquartile range (IQR)] [25], as appropriate, and categorical variables as counts and percentages. For continuous outcomes, pooled effect estimates were expressed as mean differences (MDs) with 95% confidence intervals (CIs). For studies reporting continuous variables as medians and interquartile ranges, means and standard deviations were estimated using the methods proposed by Wan et al. and Luo et al. [26,27]

For continuous outcomes measured before and after initiation of ECCO_2_R integrated with RRT, treatment effects were modeled as within-study repeated-measures changes from baseline. For each study and time point (2 h, 6 h, and 24 h), the effect size was defined as the mean change from baseline, calculated as the post-treatment mean minus the baseline mean. When the standard deviation (SD) of the change score was not directly reported, it was estimated from the baseline and follow-up SDs using the conventional formula for paired data: SD_change = √(SD_baseline^2^ + SD_follow-up^2^ − 2 r × SD_baseline × SD_follow-up), where r represents the within-subject correlation between repeated measurements. A prespecified correlation coefficient of 0.70 was assumed for the primary analysis. Study-specific standard errors were then derived from the SD of the change score and sample size. Pooled mean differences (MDs) and corresponding 95% confidence intervals (CIs) were synthesized using random-effects models with restricted maximum likelihood (REML) estimation, acknowledging anticipated clinical and methodological heterogeneity across studies. Statistical heterogeneity was assessed using Cochran’s Q test and the I^2^ statistic, with I^2^ values greater than 50% considered indicative of substantial heterogeneity. For outcomes with substantial heterogeneity, sensitivity analyses were performed using a leave-one-out approach, in which each study was sequentially excluded and the pooled effect re-estimated. Because the number of studies available for each pooled analysis was small, meta-regression was not performed, as study-level associations would have been underpowered and prone to spurious findings. Similarly, publication bias was not formally assessed with funnel plots, because such analyses are generally uninformative and potentially misleading when few studies are included. All tests were two-sided, and a p value <0.05 was considered statistically significant. All analyses were performed in R version 4.5.3 (R Foundation for Statistical Computing, Vienna, Austria).

## Results

### Study selection and characteristics

The PRISMA flow diagram of the study selection process is shown in Fig. 1. The initial database search yielded 522 records. After removal of duplicates (76), 447 records underwent title and abstract screening, of which 425 were excluded. Twenty-two full-text reports were assessed for eligibility, and 15 were excluded for the following reasons: recruitment phase [28], congress abstract (n = 3), standalone ECCO_2_R (n = 3), ECMO and/or NIV (n = 2), and review article (n = 2). Ultimately, 7 studies comprising 105 patients were included in the review [25,[28], [29], [30], [31], [32], [33]].

Baseline characteristics of the included studies are summarized in Table 1, and key technical aspects of the combined ECCO_2_R–RRT approach are detailed in Supplementary Table S2. Across studies, patients were generally older adults, predominantly male, and had moderate-to-severe hypoxemia, with baseline PaO_2_/FiO_2_ values ranging from approximately 108–163 mmHg. Severity of illness was high when reported, with SAPS II values ranging from 34 to 69 and SOFA scores from 7 to 14. Respiratory system compliance, when available, was low, ranging from approximately 23–31 mL/cmH_2_O. Both COVID-19 and non-COVID-19 ARDS populations were represented, and mortality varied substantially across studies, reflecting differences in case mix, severity, and study design.

**Table 1:** Baseline characteristics of included studies.

Variable	Allardet-Servent (2015) [28]	Nentwich (2019) [29]	Alessandri (2023) [30]	Dessap (2023) [31]	Pasero (2024) [25]	Combes (2026) [32]	Kryvenko (2026) [33]
N	11	20	27	8	14	16	9
Country	France	Germany	Italy	France	Italy	France	Germany
Diagnosis/Indication	ARDS	ARDS	ARDS	ARDS	ARDS	ARDS	ARDS
Age (years)	70 ± 9	64 (43–82)	64 ± 11	59 (37–81)	65 (62–72)	63.5 (55–69)	59.5 (53.5–67.0
Male (%)	73	60	89	63	64.3	74.1	55
Severity score (SAPS II)	69 ± 13	57 (27–79)	34 ± 14	46 (44–51)	37 (34–50)	45	66 (50–70)
SOFA	14 ± 4	14	7 ± 2	8	8.5 (6–9)	8.5 (6–11)	12 (8–14)
Lung Injury Score	3 ± 0.5	NR	NR	NR	NR	NR	NR
PaO_2_/FiO_2_	135 ± 41	159 ± 36	108 ± 29	111 (79–173)	121.5 (102−155)	163 (135–205)	158.0 (127.1–221.1)
COVID-19	No	No	Yes	No	Yes	Yes	Yes
CRS, static compliance (mL/cmH_2_O)	30 ± 9	27.5 ± 10.8	23.2 ± 2.7	NR	26.5 (20−32)	31.0 (25.0–43.0	NR
Ventilatory ratio	NR	NR	2.9 ± 1.1	NR	NR	1.7 (1.5–2.1)	NR
Creatinine (mg/dL)	3.89 ± 1.77	3.30 ± 2.59	3.30 ± 1.27	NR	NR	NR	NR
MV duration (days)	31	NR	NR	24 (16–33)	23.5	NR	NR
ICU LOS (days)	40	NR	28 ± 14	24 (18–34)	23.5	19.5 (16.5–29.5)	NR
Mortality	82%	10%	63%	38%	57%	31%	45%

Values are presented as mean ± standard deviation, median (interquartile range), or number (%), according to the format reported in the original studies. ARDS, acute respiratory distress syndrome; APACHE II, Acute Physiology and Chronic Health Evaluation II; COPD, chronic obstructive pulmonary disease; CRS, respiratory system compliance; ICU LOS, intensive care unit length of stay; MV, mechanical ventilation; NR, not reported; PaO_2_/FiO_2_, ratio of arterial oxygen partial pressure to fraction of inspired oxygen; SAPS II, Simplified Acute Physiology Score II; SOFA, Sequential Organ Failure Assessment.

With respect to renal support, continuous venovenous hemodiafiltration was the most reported CRRT modality, although prescription details were described across studies. When reported, dialysis dose ranged from fixed hourly prescriptions to weight-based regimens. Blood flow through the extracorporeal circuit was also variable across studies, ranging from approximately 186–420 mL/min, while sweep gas flow generally ranged from 2,5 to 11 L/min.

### Primary outcomes: PaCO_2_ and pH

**Fig. 2:**
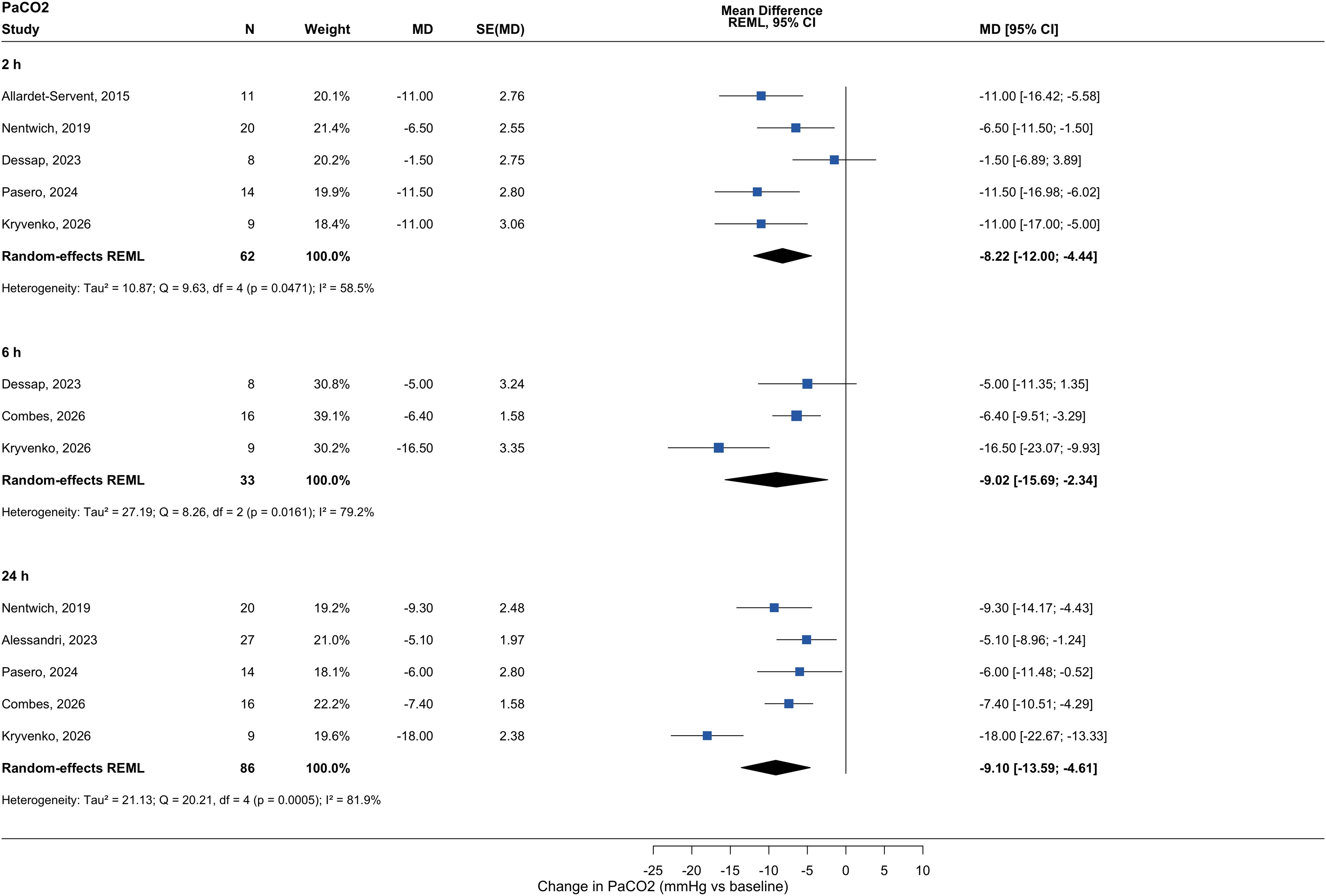
Forest plot of pooled pre–post changes in PaCO_2_ after initiation of extracorporeal CO_2_ removal. Mean differences from baseline in arterial carbon dioxide tension at 2, 6, and 24 h after initiation of ECCO_2_R-based support. Negative values indicate reductions in PaCO_2_ compared with baseline. Individual study estimates are represented by blue squares, with horizontal lines indicating 95% confidence intervals; square size reflects the relative study weight. Black diamonds represent pooled random-effects estimates using restricted maximum likelihood. Heterogeneity is reported as τ^2^, Cochran’s Q, and I^2^. MD, mean difference; SE, standard error; CI, confidence interval; REML, restricted maximum likelihood; PaCO_2_, arterial partial pressure of carbon dioxide.

**Fig. 3:**
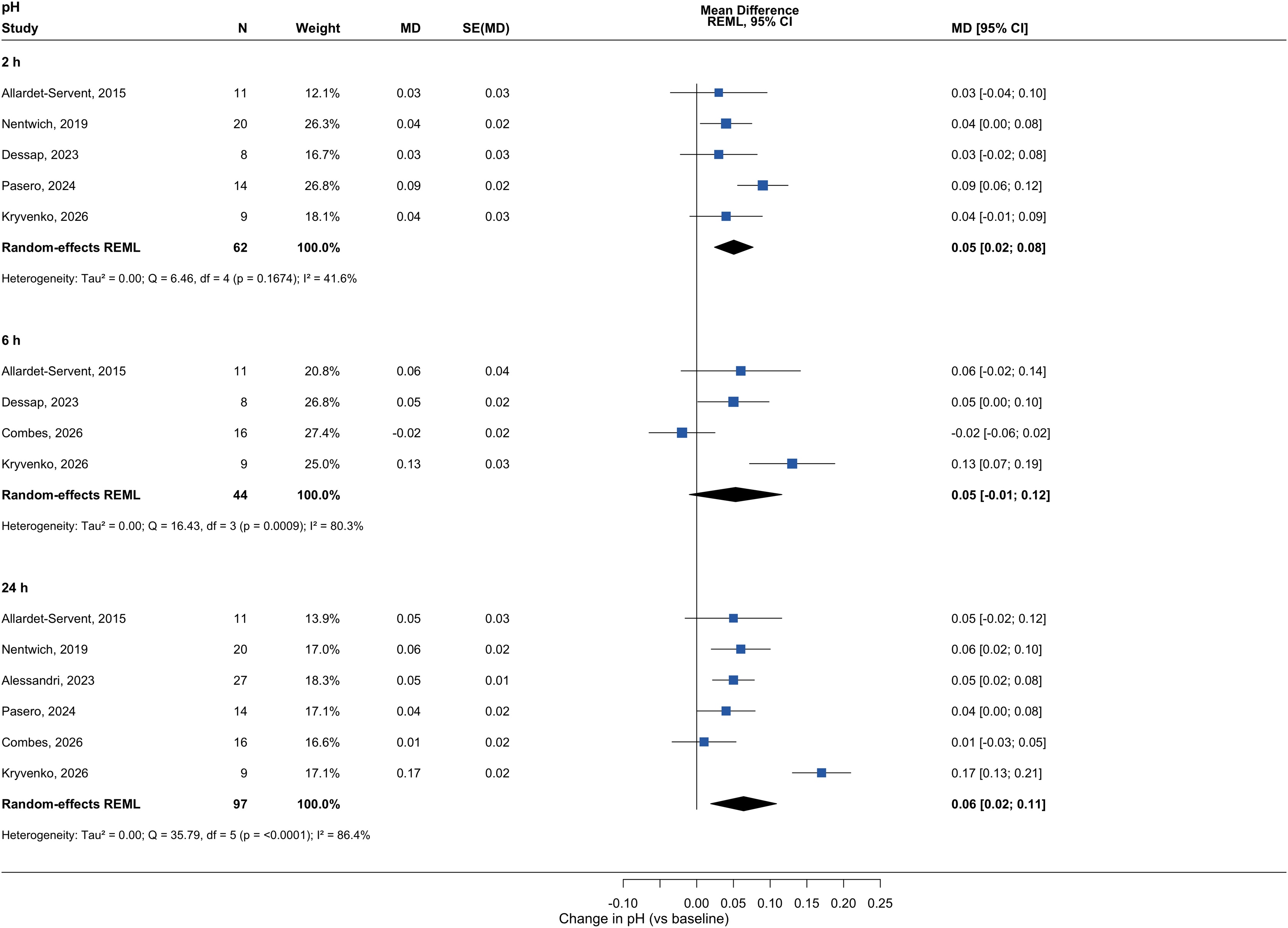
Forest plots of pooled pre–post changes in arterial pH after initiation of extracorporeal CO_2_ removal. Mean differences from baseline in arterial pH at 2, 6, and 24 h after initiation of ECCO_2_R-based support. Positive values indicate an increase in pH compared with baseline. Individual study estimates are represented by blue squares, with horizontal lines indicating 95% confidence intervals; square size reflects the relative study weight. Black diamonds represent pooled random-effects estimates using restricted maximum likelihood. Heterogeneity is reported as τ^2^, Cochran’s Q, and I^2^. MD, mean difference; SE, standard error; CI, confidence interval; REML, restricted maximum likelihood.

In pooled pre–post analyses, ECCO_2_R integrated with RRT was associated with an early and sustained reduction in PaCO_2_, with pooled mean differences of −8.22 mmHg at 2 h (95% CI −12.00 to −4.44; I^2^ = 58.5%), −9.02 mmHg at 6 h (95% CI −15.69 to −2.34; I^2^ = 79.2%), and −9.10 mmHg at 24 h (95% CI −13.59 to −4.61; I^2^ = 81.9%) (Fig. 2). Arterial pH increased at 2 h, with a pooled mean difference of +0.05 (95% CI 0.02 to 0.08; I^2^ = 41.6%), and at 24 h, with a pooled mean difference of +0.06 (95% CI 0.02 to 0.11; I^2^ = 86.4%). At 6 h, the pooled estimate suggested a smaller increase in arterial pH that did not reach statistical significance (MD + 0.05; 95% CI −0.01 to 0.12; I^2^ = 80.3%) (Fig. 3).

### Secondary outcomes: changes in ventilatory intensity and safety outcomes

**Fig. 4:**
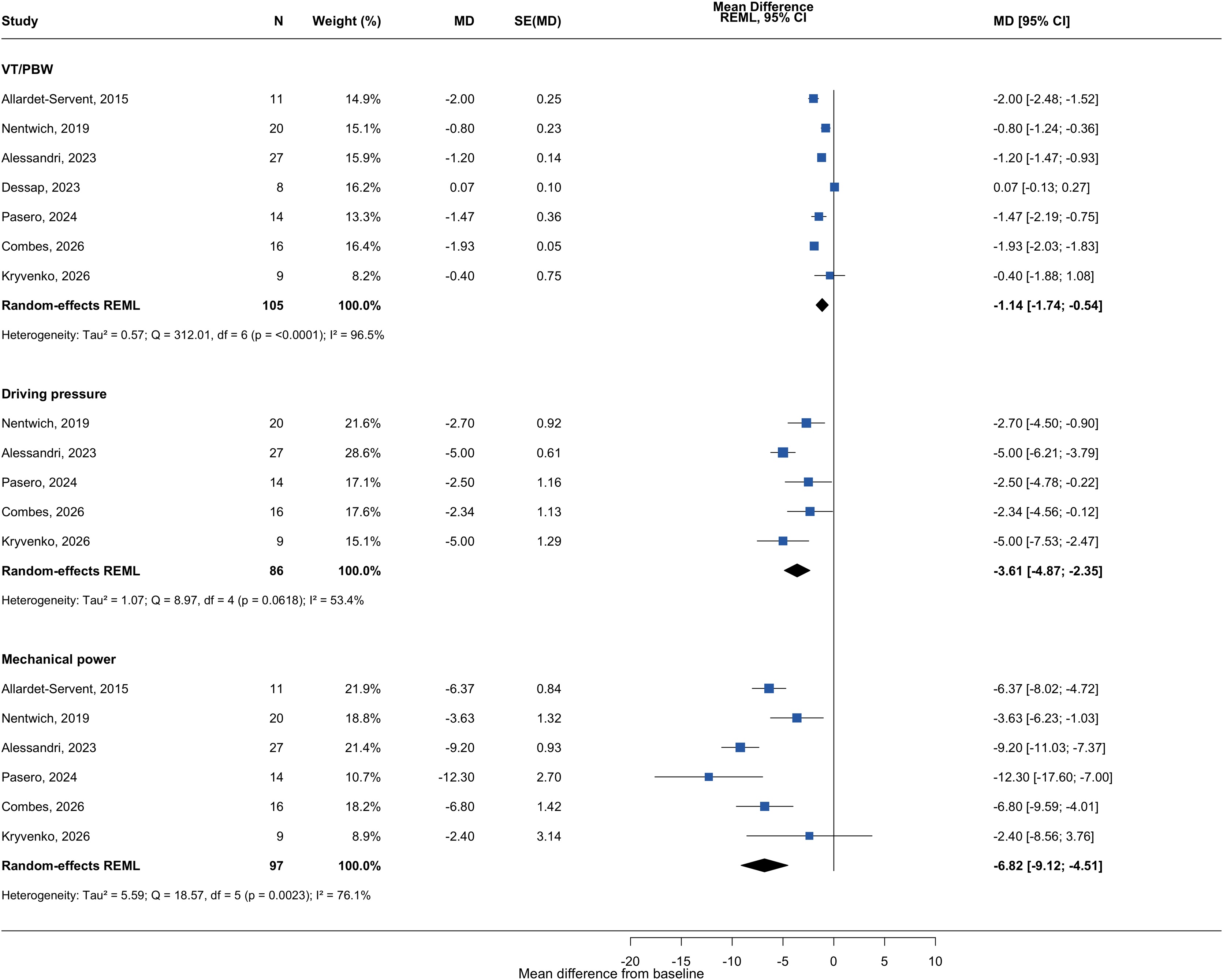
Forest plots of pooled pre–post changes in ventilatory intensity after initiation of extracorporeal CO_2_ removal. Mean differences from baseline in tidal volume normalized to predicted body weight, driving pressure, and mechanical power after initiation of ECCO_2_R-based support. Negative values indicate reductions in ventilatory intensity compared with baseline. Individual study estimates are represented by blue squares, with horizontal lines indicating 95% confidence intervals; square size reflects the relative study weight. Black diamonds represent pooled random-effects estimates using restricted maximum likelihood. Heterogeneity is reported as τ^2^, Cochran’s Q, and I^2^. VT/PBW, tidal volume normalized to predicted body weight; MD, mean difference; SE, standard error; CI, confidence interval; REML, restricted maximum likelihood.

ECCO_2_R–RRT was associated with lower ventilatory intensity. In pooled analyses, driving pressure decreased by a mean difference of −3.61 cmH_2_O (95% CI −4.87 to −2.35; I^2^ = 53.4%), mechanical power by −6.82 J/min (95% CI − 9.12 to −4.51; I^2^ = 76.1%), and tidal volume normalized to predicted body weight by −1.14 mL/kg PBW (95% CI −1.74 to −0.54; I^2^ = 96.5%) (Fig. 4). By contrast, respiratory rate did not significantly change after ECCO_2_R–RRT initiation (MD − 0.59 breaths/min; 95% CI −1.57 to 0.38; I^2^ = 41.4%). In studies with sufficient data for mechanical power decomposition, the reduction in mechanical power appeared to be primarily driven by reductions in driving pressure and tidal volume rather than by changes in respiratory rate (Supplementary Figure S4).

Reporting of safety outcomes varied across the included studies (Supplementary Table S3). Among the seven studies with available safety data, circuit-related events were the most frequently reported complications. Circuit clotting was the predominant event, occurring in 22 patients (21.0%), followed by circuit malfunction in 5 patients (4.8%) and catheter-related complications in 2 patients (1.9%). Metabolic complications were uncommon, with 1 reported event (1.0%). Bleeding was reported in 1 patient (1.0%). No clinically apparent intracranial hemorrhage, other serious adverse events, or hemolysis were reported in the studies with available safety data; however, adverse event ascertainment was heterogeneous, and the included studies did not necessarily use systematic screening protocols for these complications. Mortality was variably reported across studies, with one study reporting only 24-h mortality, limiting pooled interpretation of this outcome.

### Sensitivity analysis

Given the substantial heterogeneity across several pooled analyses, leave-one-out sensitivity analyses were performed to assess the robustness of the main findings. For PaCO_2_, the pooled reduction remained directionally consistent and statistically significant in all leave-one-out models at 2 and 24 h. At 6 h, the pooled estimate remained negative in all analyses, but the confidence interval crossed the null after exclusion of Combes et al. [32], indicating some sensitivity of this time point to individual-study influence. For arterial pH, the pooled increase remained statistically significant in all leave-one-out models at 2 and 24 h. In contrast, the 6-h pH analysis was less robust and remained inconclusive in most leave-one-out models, consistent with the non-significant overall pooled estimate at this time point. By comparison, the pooled reductions in Vt/PBW, driving pressure, and mechanical power remained directionally consistent and statistically significant in all leave-one-out models, indicating that these findings were not driven by any single study (Supplementary Figures S1–S3).

### Quality assessment and certainty of evidence

Most uncontrolled before-after studies were rated as fair quality according to the NIH Quality Assessment Tool, whereas one early proof-of-concept study was rated as poor. The only comparative non-randomized study, assessed with ROBINS-I, was judged to be at serious risk of bias, mainly because of confounding and selection-related limitations. Detailed assessments are provided in Supplementary Tables S4 and S5.

Using the GRADE framework, the certainty of evidence was judged to be very low for all outcomes. This reflected the predominance of small, uncontrolled before-after observational studies, limited causal inference due to the absence of control groups, inconsistency across studies, and imprecision due to limited sample sizes. Additional downgrading for indirectness was applied to physiological surrogate outcomes because they do not directly measure patient-centered clinical benefit. Mortality and safety outcomes were also rated as very low certainty because of heterogeneous reporting and insufficient power to assess patient-centered outcomes. Detailed certainty assessments are provided in Supplementary Table S6.

## Discussion

In this systematic review and meta-analysis of 7 studies comprising 105 patients, ECCO_2_R integrated with RRT was associated with early physiological improvement in adults with ARDS and concomitant AKI requiring continuous renal replacement therapy. Across pooled analyses, this hybrid strategy promoted rapid PaCO_2_ reduction and progressive correction of acidemia, while also allowing reductions in ventilatory intensity, including tidal volume normalized to predicted body weight, driving pressure, and mechanical power. Overall, these findings suggest that ECCO_2_R–RRT may represent a feasible extracorporeal support strategy in selected patients, with the potential to support a more protective ventilatory approach.

The relevance of these findings extends beyond carbon dioxide clearance alone. In patients with ARDS, especially when accompanied by AKI, ventilatory management often requires balancing correction of severe acidemia against the risk of ventilator-induced lung injury. In this context, the possibility of reducing ventilatory intensity while maintaining acceptable acid-base control is clinically meaningful. This interpretation is also consistent with the broader physiological literature in ARDS, in which driving pressure, respiratory rate, and mechanical power have been associated with outcome, with driving pressure showing a particularly strong relationship with mortality [4]. From a mechanistic standpoint, the expected effect of ECCO_2_R is also not uniform across patients, since dead space and respiratory system compliance are key determinants of how much extracorporeal CO_2_ removal can translate into lower driving pressure and mechanical power [34].

Our findings are directionally aligned with the contemporary ECCO_2_R literature. Broad systematic reviews have consistently shown that ECCO_2_R standalone can reduce PaCO_2_ and improve pH, while facilitating less intensive ventilation, although effects on mortality and other patient-centered outcomes remain uncertain [35,36]. More recent ARDS-focused meta-analytic data also support the ability of ECCO_2_R standalone to facilitate ultra-protective ventilation, with reductions in tidal volume, plateau pressure, and driving pressure, albeit at the cost of non-negligible device-related complications [37]. Within that context, the present study adds a more specific perspective by focusing on ECCO_2_R integrated with RRT, a combined platform that has been less frequently evaluated as a distinct strategy.

This distinction is clinically relevant because ECCO_2_R coupled with RRT is not simply a lower-flow version of standalone ECCO_2_R. Integration into a CRRT platform offers simultaneous respiratory and renal support in a population in whom acid-base homeostasis is frequently impaired by both lung and kidney dysfunction. Recent expert and consensus documents have emphasized that ARDS remains the main indication for ECCO_2_R, particularly when the objective is to enable a more protective ventilatory strategy through reductions in tidal volume, driving pressure, and respiratory rate while controlling hypercapnic acidosis [14,16,38]. These same documents also highlight the practical appeal of ECCO_2_R–RRT in patients already receiving renal support, since the technique can be implemented on familiar CRRT platforms and may not require additional vascular access beyond that already used for dialysis [16].

At the same time, these findings should be interpreted considering the mixed results of prior ECCO_2_R studies. The negative results of standalone ECCO_2_R trials, particularly REST, tempered initial enthusiasm by showing no improvement in major clinical outcomes and a higher burden of complications, especially bleeding [12,15]. However, subsequent analyses and recent reviews suggest that these results may reflect, at least in part, issues related to patient selection, extraction capacity, pump technology, anticoagulation strategy, and center experience, rather than the absence of a physiological effect per se [16,39]. In that sense, the combined ECCO_2_R–RRT platform may deserve separate consideration, particularly in patients with concomitant AKI, severe acidosis, and a clear need to reduce ventilatory stress.

The safety findings of the present review should also be interpreted within this broader framework. In our pooled dataset, reported adverse events were predominantly circuit-related, with clotting as the most frequent complication.Clinically apparent bleeding, intracranial hemorrhage, and hemolysis were uncommon or not reported; however, adverse-event ascertainment was heterogeneous and largely based on non-systematic surveillance.This pattern differs somewhat from broader ECCO_2_R meta-analyses and REST trial, in which bleeding often emerges as a leading complication [15,35,37]. Such differences likely reflect variation in device platform, blood flow range, membrane characteristics, anticoagulation protocols, and case mix. Recent data with newer roller pump-based systems also suggest that the technical profile of ECCO_2_R may differ according to platform design, with the possibility of lower hemolysis and bleeding at low flow rates, although membrane clotting remains a relevant concern [32,39].

A major strength of this study is that it specifically addresses ECCO_2_R integrated with RRT as a combined extracorporeal support strategy, rather than ECCO_2_R used in isolation across heterogeneous clinical contexts. In addition, the present analysis extends beyond gas exchange by incorporating ventilatory variables with recognized physiological relevance, particularly driving pressure and mechanical power. Nevertheless, the limitations of the available evidence remain substantial. The number of studies was small, most reports were non-randomized, and important heterogeneity was present on patient selection, extracorporeal configuration, ventilatory management, and timing of assessment. Because most included studies lacked a control group, the observed changes in PaCO_2_, pH, and ventilatory variables cannot be attributed causally to ECCO_2_R–RRT, as co-interventions and time-varying clinical management may have contributed to these findings. In addition, leave-one-out analyses assessed the influence of individual studies on the pooled estimates but did not explain the substantial between-study heterogeneity, which likely reflects differences in patient selection, disease severity, device configuration, blood flow, sweep gas flow, CRRT prescription, ventilatory protocols, and timing of outcome assessment.Moreover, most studies were designed to evaluate short-term physiological responses rather than patient-centered outcomes, which remained inconsistently reported. Accordingly, the present findings should be interpreted as supportive of physiological feasibility rather than definitive evidence of clinical benefit.

## Conclusion

ECCO_2_R integrated with RRT was associated with early PaCO_2_ reduction, improvement in pH, and lower ventilatory intensity in patients with ARDS and concomitant AKI. These findings suggest that ECCO_2_R–RRT supports physiological feasibility to facilitate more protective ventilation in selected patients. However, the current evidence remains limited and heterogeneous, and larger prospective controlled studies are required to establish its clinical role and impact on patient-centered outcomes.

## Authors’ contributions

YAPS and PRGL conceived the study idea and defined the review objectives and methodology. YAPS, PRGL, BMT and FJSR, conducted the systematic literature search and independently extracted data from eligible studies. YAPS, BMT, MCC, LPJ, and AC performed the statistical analysis and meta-analytical synthesis. YAPS, ELVC, MCC, LPJ, MBA and AC contributed to the interpretation of the results and the assessment of study quality. YAPS, PRGL drafted the initial manuscript. All authors critically revised the manuscript for important intellectual content, contributed to the final text, and approved the submitted version.

## Consent for publication

Not applicable. This manuscript does not contain any individual person’s data in any form.

## Ethics approval and consent to participate

Not applicable. This study is a systematic review and meta-analysis of previously published clinical cohorts and/or randomized controlled trials and did not involve access to individual patient data.

## Funding

This research received no specific grant from any funding agency in the public, commercial, or not-for-profit sectors. No institutional or departmental funds were used.

## Availability of data and materials

All data generated or analyzed during this study are included in this published article and its supplementary information files. The review protocol was prospectively registered in PROSPERO (CRD420251234033).

## Declaration of competing interest

PRGL is an employee of Vantive Health. This relationship had no role in the design, conduct, analysis, or reporting of the present study. The other authors declare that they have no competing interests.
