# Supplementary material for: Extracorporeal CO2 removal integrated with continuous renal replacement therapy in patients with acute respiratory distress syndrome and acute kidney injury: a systematic review and meta-analysis
Source: Ann Intensive Care. 2026 Jul 15;16:100114. doi: 10.1016/j.aicoj.2026.100114 (PMC13396961; doi:10.1016/j.aicoj.2026.100114)

**Extracorporeal CO₂ Removal Integrated with Continuous Renal Replacement Therapy in Patients with Acute Respiratory Distress Syndrome and Acute Kidney Injury: A Systematic Review and Meta-analysis**

**Supplementary Material**

Summary

[Supplementary Table S1 – Search Strategy 2](#_Toc233831416)

[Supplementary Table S2 – Extracorporeal support configuration, renal replacement therapy prescription, and ventilatory management strategies across the included studies. 3](#_Toc233831417)

[Supplementary Table S3 – Safety outcomes across the included studies 4](#_Toc233831418)

[Supplementary Table S4 – NIH Quality Assessment Tool for Before-After (Pre-Post) Studies With No Control Group. 5](#_Toc233831419)

[Supplementary Table S5 – Risk of Bias Assessment - ROBINS-1 6](#_Toc233831420)

[Supplementary Table S6 – GRADE Assessment of Certainty of Evidence 7](#_Toc233831421)

[Supplementary Figure S1. Leave-one-out sensitivity analyses of changes in PaCO₂ after initiation of ECCO₂R integrated with RRT 9](#_Toc233831422)

[Supplementary Figure S2. Leave-one-out sensitivity analyses of changes in pH after initiation of ECCO₂R integrated with RRT. 10](#_Toc233831423)

[Supplementary Figure S3. Leave-one-out sensitivity analyses of changes in ventilatory mechanics after initiation of ECCO₂R integrated with RRT 11](#_Toc233831424)

| **Pubmed** | ( "Respiratory Distress Syndrome, Adult"[Mesh] OR "acute respiratory distress syndrome"[tiab] OR ARDS[tiab] OR "Chronic Obstructive Pulmonary Disease"[Mesh]  OR COPD[tiab] OR "chronic obstructive"[tiab] OR "acute exacerbation"[tiab] OR "acute respiratory failure"[tiab] OR "respiratory failure"[Mesh] OR hypercapnia[tiab]  OR hypercapnic[tiab] OR "ventilatory failure"[tiab] OR "CO2 retention"[tiab])  AND ( "Acute Kidney Injury"[Mesh] OR "acute kidney injury"[tiab] OR AKI[tiab] OR "kidney failure, acute"[Mesh] OR "renal failure acute"[tiab] OR "renal replacement therapy"[tiab] OR "renal replacement therapy"[Mesh] OR CRRT[tiab]  OR RRT[tiab] OR CVVH[tiab] OR CVVHD[tiab] OR CVVHDF[tiab] OR hemofiltration[tiab] OR hemodiafiltration[tiab]) AND ("Extracorporeal Carbon Dioxide Removal"[Mesh] OR "extracorporeal CO2 removal"[tiab] OR ECCO2R[tiab]  OR "extracorporeal CO2"[tiab] OR "carbon dioxide removal"[tiab]  OR "low-flow CO2 removal"[tiab] OR "membrane lung"[tiab] OR oxygenator[tiab] OR "gas exchange membrane"[tiab] OR "artificial lung"[tiab] OR "in-line oxygenator"[tiab]  OR "integrated oxygenator"[tiab] OR "CRRT circuit"[tiab] OR "RRT circuit"[tiab] OR "hybrid extracorporeal"[tiab] OR "renal pulmonary support"[tiab]  OR "combined extracorporeal"[tiab] OR "CO2 dialysis"[tiab]) |
| --- | --- |
| **EMBASE** | ('acute respiratory distress syndrome'/exp OR 'acute respiratory distress syndrome':ti,ab,kw OR 'ards':ti,ab,kw OR 'chronic obstructive lung disease'/exp OR 'copd':ti,ab,kw OR 'chronic obstructive':ti,ab,kw OR 'acute exacerbation':ti,ab,kw OR 'acute respiratory failure':ti,ab,kw OR 'respiratory failure'/exp OR 'hypercapnia':ti,ab,kw OR 'hypercapnic':ti,ab,kw OR 'ventilatory failure':ti,ab,kw OR 'co2 retention':ti,ab,kw) AND ('acute kidney failure'/exp OR 'acute kidney injury':ti,ab,kw OR 'aki':ti,ab,kw OR 'acute kidney failure'/exp OR 'renal failure acute':ti,ab,kw OR 'renal replacement therapy':ti,ab,kw OR 'renal replacement therapy'/exp OR 'crrt':ti,ab,kw OR 'rrt':ti,ab,kw OR 'cvvh':ti,ab,kw OR 'cvvhd':ti,ab,kw OR 'cvvhdf':ti,ab,kw OR 'hemofiltration':ti,ab,kw OR 'hemodiafiltration':ti,ab,kw) AND ('extracorporeal carbon dioxide removal'/exp OR 'extracorporeal co2 removal':ti,ab,kw OR 'ecco2r':ti,ab,kw OR 'extracorporeal co2':ti,ab,kw OR 'carbon dioxide removal':ti,ab,kw OR 'low-flow co2 removal':ti,ab,kw OR 'membrane lung':ti,ab,kw OR 'oxygenator':ti,ab,kw OR 'gas exchange membrane':ti,ab,kw OR 'artificial lung':ti,ab,kw OR 'in-line oxygenator':ti,ab,kw OR 'integrated oxygenator':ti,ab,kw OR 'crrt circuit':ti,ab,kw OR 'rrt circuit':ti,ab,kw OR 'hybrid extracorporeal':ti,ab,kw OR 'renal pulmonary support':ti,ab,kw OR 'combined extracorporeal':ti,ab,kw OR 'co2 dialysis':ti,ab,kw) |
| **Cochrane Library** | ("respiratory distress syndrome" OR ARDS OR "acute respiratory distress syndrome" OR "acute respiratory failure" OR "respiratory failure" OR hypercapnia OR hypercapnic OR "CO2 retention" OR "ventilatory failure" OR "chronic obstructive pulmonary disease" OR COPD)  AND("acute kidney injury" OR AKI OR "acute renal failure" OR "renal replacement therapy" OR RRT OR CRRT OR CVVH OR CVVHD OR CVVHDF OR hemofiltration OR hemodiafiltration)AND  ( "extracorporeal CO2 removal" OR ECCO2R OR "extracorporeal carbon dioxide removal" OR "CO2 removal" OR "low-flow CO2 removal" OR "membrane lung" OR "gas exchange membrane" OR oxygenator OR "in-line oxygenator" OR "integrated oxygenator" OR "artificial lung" OR "CO2 dialysis" OR "CRRT circuit" OR "RRT circuit" OR "combined extracorporeal" OR "renal pulmonary support" OR "hybrid extracorporeal" OR "extracorporeal CO2") |

# **Supplementary Table S1 – Search Strategy**

# **Supplementary Table S2 – Extracorporeal support configuration, renal replacement therapy prescription, and ventilatory management strategies across the included studies.**

| **Variable** | **Allardet-Servent (2015)** | **Nentwich**  **(2019)** | **Alessandri (2023)** | **Dessap**  **(2023)** | **Pasero**  **(2024)** | **Combes**  **(2026)** | **Kryvenko**  **(2026)** |
| --- | --- | --- | --- | --- | --- | --- | --- |
| **ECCO2R Membrane** | Prismalung | Prismalung | Eurosets | Prismalung | Prismalung+ or OMNI | Prismalung+ | multiECCO_2_R |
| **CRRT machine** | Prismaflex | Prismaflex | OMNI | Prismaflex | PrisMax or OMNI | PrisMax | Multifiltrate |
| **ECCO_2_R filter area (m2)** | 0.32 | 0.32 | 1.81 | 0.23 | 0.80 | 0.80 | 1.35 |
| **CRRT filter area (m2)** | 1.50 | NR | 1.60 | 1.40 | 1.50 | 1.50 | 1.8 |
| **CRRT modality** | CVVHDF | CVVHDF | 15 CVVHDF / 6 CVVH / 6 CVVHD | CVVHDF | CVVHDF | CVVHDF | CVVHDF |
| **CRRT dose** | 46 (39 - 54) mL/Kg/H | 2615 ± 470 mL/H | NR | NR | NR | NR | NR |
| **BFR (mL/min)** | 420.0 | 400.0 | 186 - 393 | 300 (275 - 300) | 365.0 | 350 (300 - 400) | 130-214 |
| **Sweep gas (L/min)** | 8.0 | 8.0 | 9 - 11 | 10 (9.5 - 10) | 10.0 | 10.0 | 2.5 |
| **ECCO_2_R position** | PRE / POS | POS | PRE | POS | POS | POS | POS |

Extracorporeal support configuration, renal replacement therapy prescription, and ventilatory management strategies across the included studies. Data are presented as reported in the original studies and include circuit configuration, membrane characteristics, blood flow, sweep gas flow, renal replacement therapy modality, anticoagulation strategy, dialysis dose, replacement fluid strategy, membrane position relative to the hemofilter, and key ventilatory settings when available. Abbreviations: CRRT, continuous renal replacement therapy; CVVH, continuous venovenous hemofiltration; CVVHD, continuous venovenous hemodialysis; CVVHDF, continuous venovenous hemodiafiltration; D, dialysis dose; ECCO₂R, extracorporeal carbon dioxide removal; FiO₂, fraction of inspired oxygen; NR, not reported; PBS, pre-filter replacement fluid; PEEP, positive end-expiratory pressure; Pos, ECCO₂R membrane positioned after the hemofilter; Post, post-filter replacement fluid; Pre, ECCO₂R membrane positioned before the hemofilter; RRT, renal replacement therapy; Vt, tidal volume.

# **Supplementary Table S3 – Safety outcomes across the included studies**

| **Study** | **n** | **Mortality 28d** | **Bleeding** | **ICH/SAE** | **Catheter complication** | **Circuit clotting** | **Circuit malfunction** | **Hemolysis** | **Metabolic complication** |
| --- | --- | --- | --- | --- | --- | --- | --- | --- | --- |
| **Allardet- Servent**  **2015** | 11 | 9 (81.8%) | 0 (0.0%) | 0 (0.0%) | 1 (9.1%) | 1 (9.1%) | 1 (9.1%) | 0 (0.0%) | 0 (0.0%) |
| **Nentwich**  **2019** | 20 | 2 (10.0%) at 24 h only | 0 (0.0%) | 0 (0.0%) | 0 (0.0%) | 5 (25.0%) | 0 (0.0%) | 0 (0.0%) | 0 (0.0%) |
| **Alessandri**  **2023** | 27 | 17 (63.0%) | 0 (0.0%) | 0 (0.0%) | 0 (0.0%) | 3 (11.1%) | 3 (11.1%) | 0 (0.0%) | 0 (0.0%) |
| **Dessap**  **2023** | 8 | 3 (37.5%) | 0 (0.0%) | 0 (0.0%) | 1 (12.5%) | 5 (62.5%) | 0 (0.0%) | 0 (0.0%) | 1 (12.5%) |
| **Pasero**  **2024** | 14 | 8 (57.1%) | 0 (0.0%) | 0 (0.0%) | 0 (0.0%) | 2 (14.3%) | 1 (7.1%) | 0 (0.0%) | 0 (0.0%) |
| **Combes**  **2026** | 16 | 5 (31%) | 0 (0.0%) | 0 (0.0%) | 0 (0.0%) | 4 (25 %) | 0 (0.0%) | 0 (0.0%) | 0 (0.0%) |
| **Kryvenko**  **2026** | 9 | 5 (45%) | 1 (11%) | 0 (0.0%) | 0 (0.0%) | 2 (22%) | 0 (0.0%) | 0 (0.0%) | 0 (0.0%) |

Reported mortality and safety outcomes across the included studies evaluating extracorporeal carbon dioxide removal integrated with renal replacement therapy. Data are presented as number of events and corresponding percentage of the study population, calculated according to the total number of patients included in each study. Reported outcomes include 28-day mortality, bleeding, intracranial hemorrhage or other serious adverse events, catheter-related complications, circuit clotting, circuit malfunction, hemolysis, and metabolic complications. Mortality data for Nentwich et al. were available only for the first 24 h, and longer-term mortality was not reported. Abbreviations: ECCO₂R, extracorporeal carbon dioxide removal; ICH, intracranial hemorrhage; NR, not reported; SAE, serious adverse event.

# **Supplementary Table S4 – NIH Quality Assessment Tool for Before-After (Pre-Post) Studies With No Control Group.**

| **Study** | **Design** | **Overall rating** | **Main limitation** |
| --- | --- | --- | --- |
| **Allardet-Servent 2015** | Prospective observational study | Fair | Small single-center study without a comparator. |
| **Nentwich 2019** | Multicenter observational pilot study | Fair | Small sample, no control group, limited external validity. |
| **Dessap 2024** | Prospective pilot study | Fair | Early termination, very small sample, frequent membrane clotting. |
| **Alessandri 2023** | Retrospective multicenter observational study | Fair | Retrospective design, no comparator, incomplete-record exclusion risk. |
| **Combes 2026** | Prospective multicenter single-arm study | Fair | Single-arm design; , no control group |
| **Kryvenko 2026** | Prospective single-center observational before-after study | Fair | Small single-center study without a control group. |

# **Supplementary Table S5 – Risk of Bias Assessment - ROBINS-1**

| **Study** | **Confounding** | **Selection** | **Classification** | **Deviations** | **Missing data** | **Outcome measurement** | **Reported result** | **Overall** |
| --- | --- | --- | --- | --- | --- | --- | --- | --- |
| Pasero 2024 | Serious | Serious | Low | Moderate | Low | Low | Moderate | Serious |

ROBINS-I, Risk Of Bias In Non-randomized Studies - of Interventions.

# **Supplementary Table S6 – GRADE Assessment of Certainty of Evidence**

| **Outcome** | **Studies** | **Certainty** | **Reasons for downgrading** | **Plain-language interpretation** |
| --- | --- | --- | --- | --- |
| **PaCO₂ reduction** | 7 studies | ⊕◯◯◯ Very low | Very serious risk of bias due to uncontrolled before-after designs and absence of control groups; inconsistency across devices, protocols, and populations; indirectness because PaCO₂ is a physiological surrogate endpoint; imprecision due to small sample sizes. | ECCO₂R plus RRT may reduces PaCO₂, although the magnitude of effect remains uncertain. |
| **pH improvement** | 7 studies | ⊕◯◯◯ Very low | Very serious risk of bias due to uncontrolled before-after designs and limited causal inference; inconsistency; indirectness because arterial pH is a physiological surrogate endpoint; imprecision due to small sample sizes. | ECCO₂R plus RRT may improves arterial pH, although confidence in the estimate is very limited. |
| **Driving pressure reduction** | 5 studies | ⊕◯◯◯ Very low | Very serious risk of bias due to uncontrolled before-after designs; inconsistency related to ventilatory protocols and case mix; indirectness because driving pressure is a surrogate physiological endpoint; imprecision due to few small studies. | The intervention may reduces driving pressure, although the exact magnitude of benefit remains uncertain. |
| **Tidal volume reduction (VT/PBW)** | 7 studies | ⊕◯◯◯ Very low | Very serious risk of bias due to uncontrolled before-after designs; inconsistency related to ventilatory protocols and case mix; indirectness because driving pressure is a surrogate physiological endpoint; imprecision due to few small studies. | The intervention may facilitates ultraprotective ventilation, although certainty is very limited. |
| **Mechanical power reduction** | 6 studies | ⊕◯◯◯ Very low | Very serious risk of bias due to uncontrolled before-after designs; inconsistency related to ventilatory protocols and case mix; indirectness because driving pressure is a surrogate physiological endpoint; imprecision due to few small studies. | Mechanical power may decreases, although the true effect remains uncertain. |
| **Mortality** | 7 studies | ⊕◯◯◯ Very low | Risk of bias; very serious imprecision; studies were not designed or powered to detect differences in mortality. | No reliable conclusion can be drawn regarding mortality. |
| **Safety / adverse events** | 7 studies | ⊕◯◯◯ Very low | Risk of bias; inconsistency in adverse-event definitions and reporting; imprecision; lack of systematic surveillance for complications. | The safety profile remains uncertain despite several reports suggesting feasibility. |

*ECCO₂R, extracorporeal carbon dioxide removal; RRT, renal replacement therapy; VT/PBW, tidal volume normalized to predicted body weight*

# **Supplementary Figure S1**. **Leave-one-out sensitivity analyses of changes in PaCO₂ after initiation of ECCO₂R integrated with RRT**

Sequential leave-one-out analyses were performed for PaCO₂ at 2 h, 6 h, and 24 h by excluding one study at a time and re-estimating the pooled effect. The pooled reduction in PaCO₂ remained significant at 24 h across all leave-one-out models, whereas the estimates at 2 h and 6 h were more sensitive to exclusion of individual studies. Squares represent pooled mean differences (MDs) obtained after omission of each study, horizontal lines indicate 95% confidence intervals (CIs), and diamonds represent the overall random-effects estimate using restricted maximum likelihood (REML).


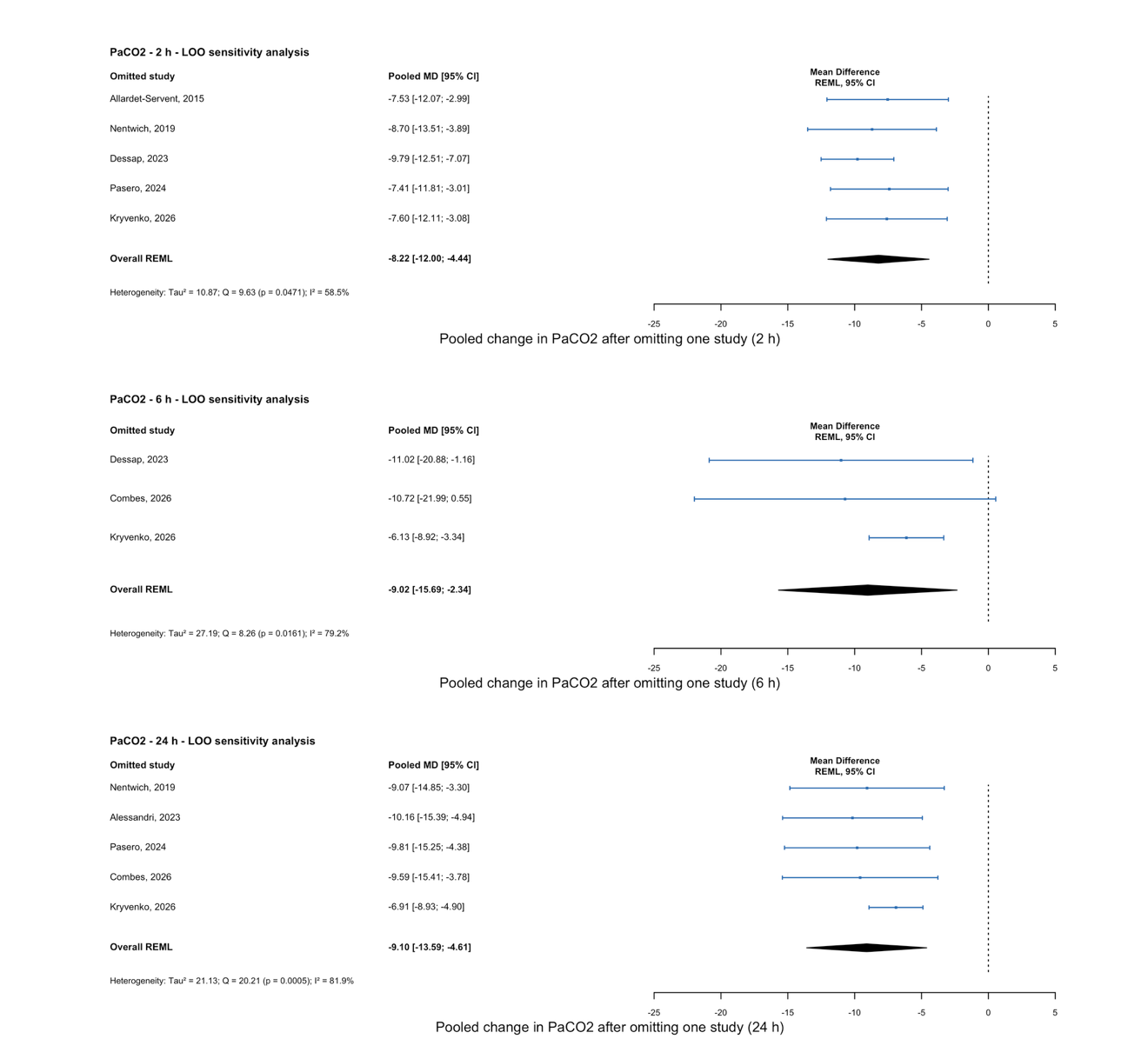


# **Supplementary Figure S2. Leave-one-out sensitivity analyses of changes in pH after initiation of ECCO₂R integrated with RRT.**

Sequential leave-one-out analyses were performed for pH at 2 h, 6 h, and 24 h by excluding one study at a time and re-estimating the pooled effect. The pooled increase in pH remained significant at 24 h across all leave-one-out models, whereas the estimates at 2 h and especially at 6 h were more sensitive to exclusion of individual studies. Squares represent pooled mean differences (MDs) obtained after omission of each study, horizontal lines indicate 95% confidence intervals (CIs), and diamonds represent the overall random-effects estimate using restricted maximum likelihood (REML).


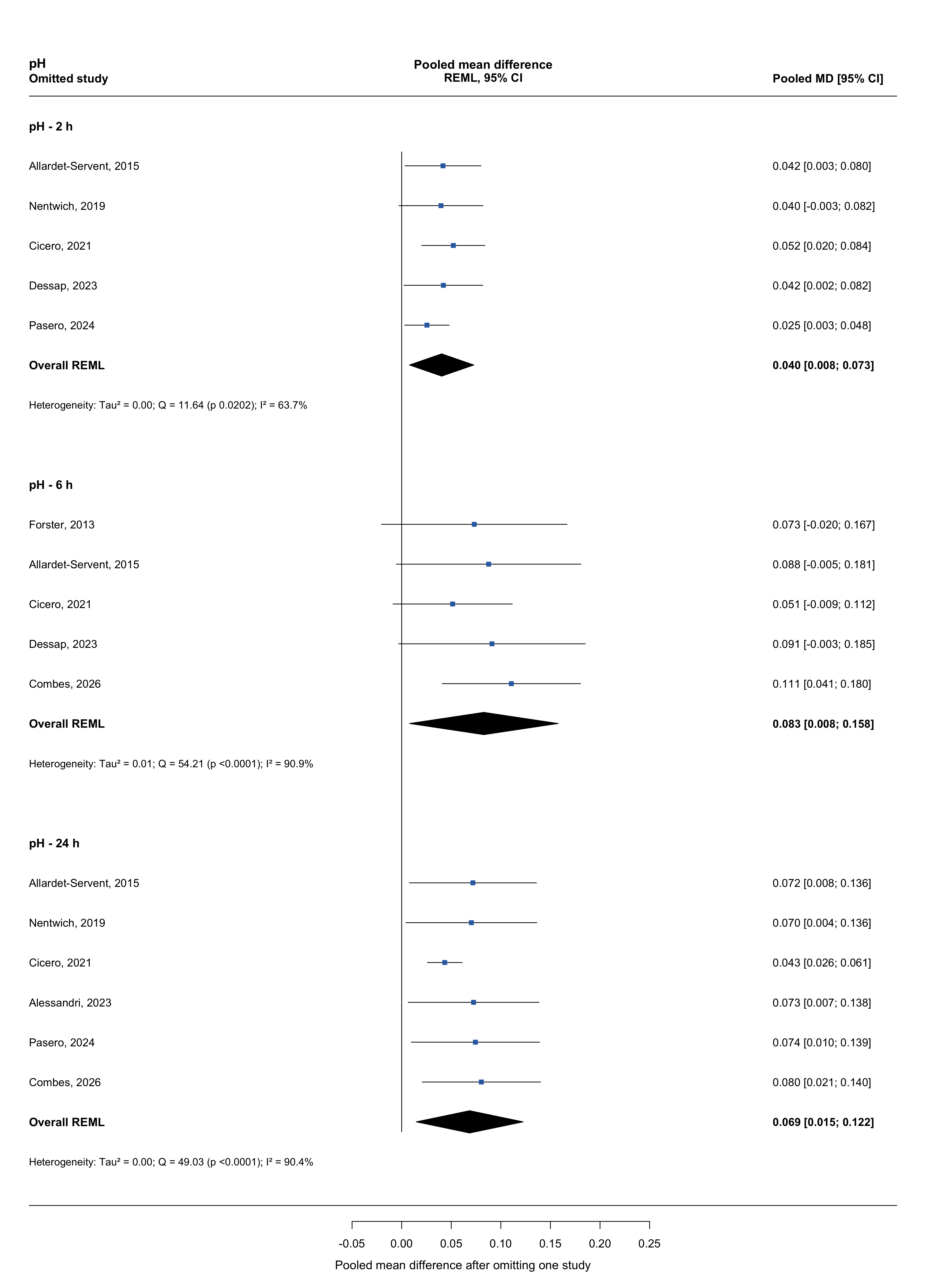


# **Supplementary Figure S3.** **Leave-one-out sensitivity analyses of changes in ventilatory mechanics after initiation of ECCO₂R integrated with RRT**

Sequential leave-one-out analyses were performed for tidal volume normalized to predicted body weight (Vt/PBW), driving pressure, and mechanical power by excluding one study at a time and re-estimating the pooled effect. The pooled effects remained directionally consistent and statistically significant across all leave-one-out models for all three outcomes, supporting the robustness of the main findings. Squares represent pooled mean differences (MDs) obtained after omission of each study, horizontal lines indicate 95% confidence intervals (CIs), and diamonds represent the overall random-effects estimate using restricted maximum likelihood (REML).


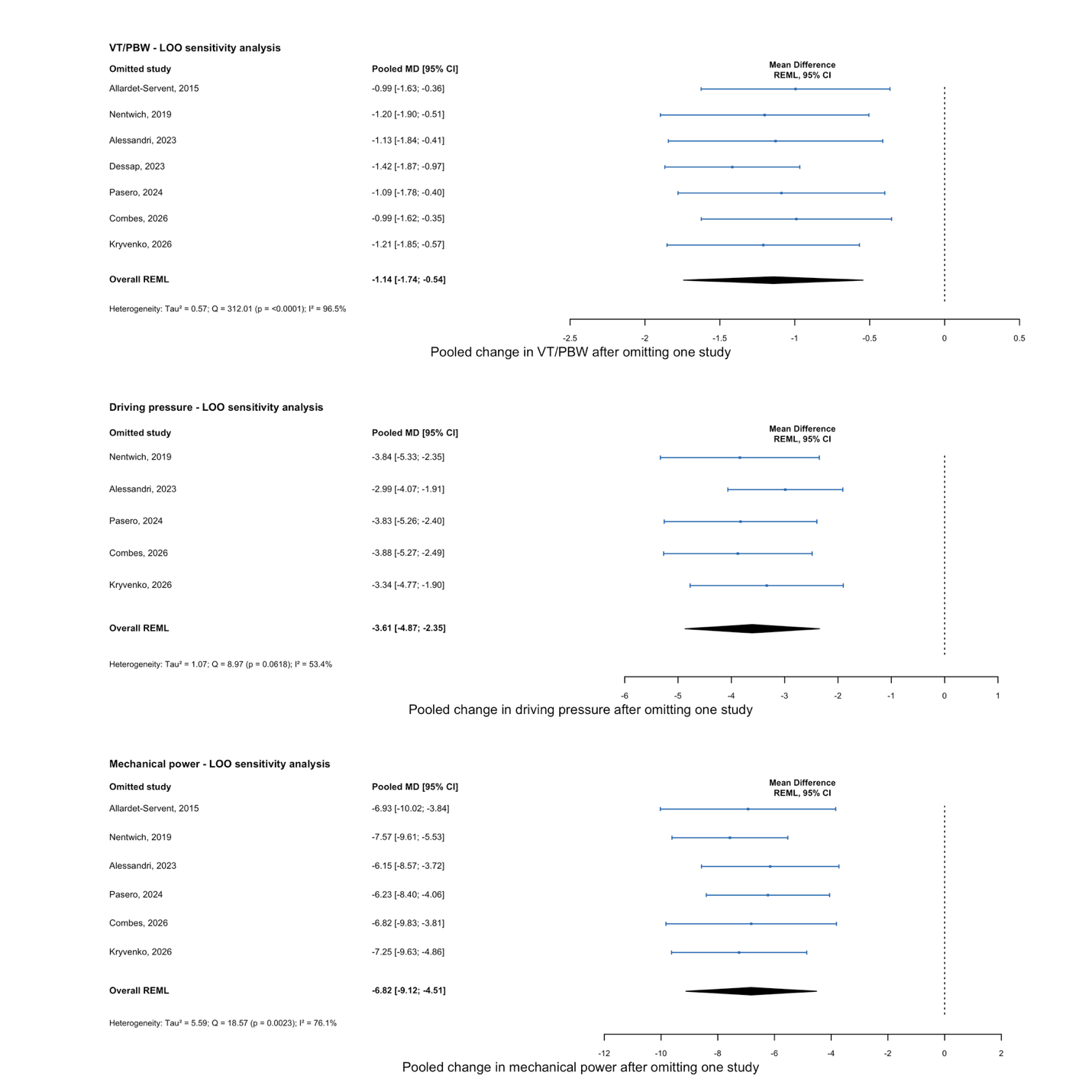


# **Supplementary Figure S4. Exploratory sequential decomposition of the reduction in mechanical power following ECCO₂R.**

Left panels show the decomposition for each individual study with sufficient ventilatory data; the right panel shows the pooled estimate. Bars represent the sequential contribution of changes in tidal volume (VT), driving pressure (ΔP), respiratory rate (RR), and positive end-expiratory pressure (PEEP) to the overall reduction in mechanical power.


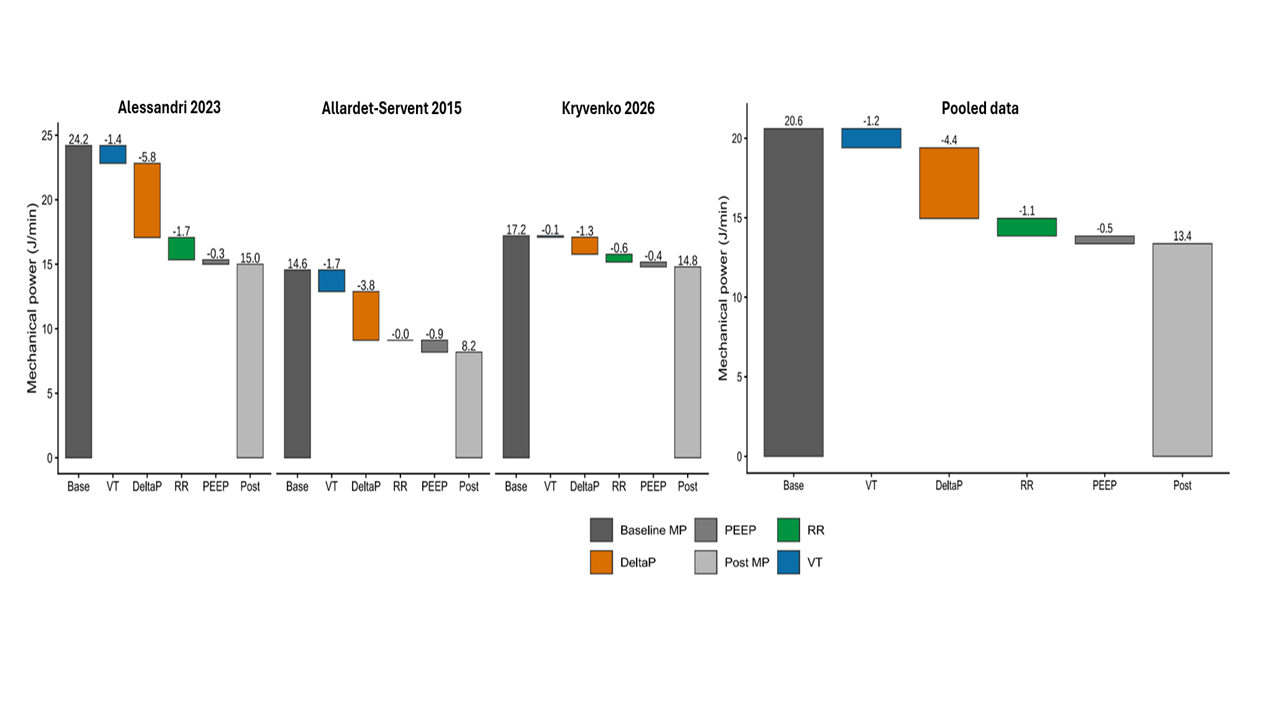

Supplement: Supplementary file 1 [file mmc1.docx]
